# Transfer to community and prison mental health care from Ireland’s main remand prison over three years: 2015-2017

**DOI:** 10.3389/fpsyt.2024.1392072

**Published:** 2024-07-19

**Authors:** Jamie Walsh, Damian Smith, Fintan Byrne, Philip Hickey, Enda Taylor, Martin Caddow, Orla Reynolds, Conor O’Neill

**Affiliations:** ^1^ National Forensic Mental Health Service, Central Mental Hospital, Dublin, Ireland; ^2^ Department of Psychiatry, Trinity College, Dublin, Ireland; ^3^ Mayo Mental Health Service, Mayo University Hospital, Mayo, Ireland; ^4^ HAIL (Housing Association for Integrated Living), Dublin, Ireland

**Keywords:** prison, mental health, social, homeless, continuity of care, transition, release planning, diversion

## Abstract

**Background:**

The post-release period is associated with an increased risk of morbidity and mortality. Previous studies have identified deficits in pre-release planning for mentally ill people in prison, particularly in remand settings.

**Objectives:**

We aimed to determine the proportion of mentally ill people in Ireland’s main remand prison who were referred for mental health follow up in community and prison settings, who achieved face to face contact with the receiving service.

**Method:**

This retrospective observational cohort study was based in Ireland’s main male remand prison, Cloverhill. Participants included all those individuals on the caseload of the prison inreach mental health team who were referred for mental health follow up in community and prison settings at the time of discharge, prison transfer or release from custody over a three-year period, 2015 - 2017. Successful transfer of care (TOC) was defined as face-to-face contact with the receiving service, confirmed by written correspondence or by follow up telephone call. Clinical, demographic and offence related variables were recorded for all participants.

**Results:**

There were 911 discharges from the prison inreach mental health team within the three-year study period. Of these, 121 were admitted to hospital, 166 were transferred to other prison inreach mental health services and 237 were discharged to community based mental health follow up in psychiatric outpatient or primary care settings. One third (304/911) had an ICD-10 diagnosis of schizophreniform or bipolar disorder (F20–31) and 37.5% (161/911) were homeless. Over 90% (152/166) of those referred to mental health teams in other prisons achieved successful TOC, with a median of six days to first face-to face assessment. Overall, 59% (140/237) of those referred to community psychiatric outpatient or primary care services achieved TOC following referral on release from custody, with a median of nine days from release to assessment. Clinical and demographic variables did not differ between those achieving and not achieving successful TOC, other than having had input from the PICLS Housing Support Service.

**Conclusion:**

Successful transfer of care can be achieved in remand settings using a systematic approach with an emphasis on early and sustained interagency liaison and clear mapping of patient pathways. For incarcerated individuals experiencing homelessness and mental health disorders, provision of a housing support service was associated with increased likelihood of successful transfer of care to community mental health supports.

## Introduction

The prevalence of major mental illness is higher among incarcerated people when compared with that of the general population (1). These individuals experience greater difficulty accessing healthcare both within prison (2) and on release to the community (3).The post-release period is associated with an increased risk of morbidity and mortality (4, 5) yet there is limited information available in the literature about clinical outcomes for people on prison mental health caseloads following release from custody or prison transfer.

On 1^st^ January 2015, the prison population of Irish prisons was 3,546. Of these, 97% (3427) were male. Of the 425 male prisoners on remand (pre-trial) on that date, 57% (244) were located in Cloverhill Prison. Cloverhill, a male-only prison, is Ireland’s main remand facility and receives a majority of remands from Ireland’s courts including those in and around Dublin, Ireland’s most populous conurbation. During the period 2012 to 2017, the prison received a majority of all remands nationally (6). The remaining remands nationally were mainly committed to three other mixed remand and sentenced facilities in the south and west of the country. A multidisciplinary mental health team has been provided 5 days a week (Monday–Friday) to the prison since 2006 to enable systematic identification and diversion to healthcare of incarcerated persons with mental illness. The team is called the Prison In-reach and Court Liaison Service (PICLS). PICLS Cloverhill is a part of the National Forensic Mental Health Service (NFMHS), a specialist tertiary mental health service funded and managed by the state health service, the Health Service Executive (HSE).

The PICLS service model, has been described in previous publications (7, 8). The team provides “inreach” by operating within the prison, delivering daily clinics as well as providing a liaison service to the adjacent District Court (equivalent to Magistrates Court in the UK) and other District Courts. The service engages with prison and NFMHS governance structures. The model of care involves a two-stage screening process supplemented by referrals from primary care and other stakeholders of all new committals to the prison. Persons thus identified as requiring urgent assessment or as vulnerable are placed on a high-support landing known as D2, which has psychiatric input five days weekly. Persons identified through screening are assessed by pairs of interviewers and a case summary is then prepared based on interview and collateral from multiple sources obtained with the patient’s consent. For each person seen, a care plan is developed to identify and arrange care pathways in the event of custodial and non-custodial disposal with early communication with agencies to whom they may be referred. Patients receive regular reviews by pairs of interviewers while they remain on the PICLS caseload. Psychiatric reports to advise regarding diversion options in forensic and community settings are prepared voluntarily and on request for District and other courts. On, or prior to release or transfer, the service communicates in writing with the receiving primary care and mental health services. During the study period and subsequently, the team consisted of a Consultant Psychiatrist, two to three psychiatric trainees, three forensic mental health nurses, and a housing support worker employed through HAIL (Housing Association for Integrated Living) from January 2014. This latter post was funded initially by the Genio Trust, an international philanthropic organisation based in Ireland, which aimed to support social services and provide innovative solutions to complex health and social care problems (9), and latterly by the HSE. Not all posts were filled throughout the study period.

Ireland has limited legislation covering court diversion for incarcerated individuals on remand. Persons found unfit to be tried under section 4 of the Criminal Law (Insanity) Act 2006 may be transferred to beds “designated” by this Act at the Central Mental Hospital (Ireland’s only inpatient forensic facility during the years studied). In part due to very limited forensic bed numbers, there were few such transfers during the three years studied. In practice, where admission beds were required, this was more frequently achieved through transfer to general community psychiatric beds when the Court granted a person bail following receipt of a report from the PICLS service, followed by use of the civil Mental Health Act 2001 (10).

Mental health services in prisons can struggle to organise community mental health follow up for their patients on their release to the community. This is particularly the case in remand settings where time spent in custody can be brief, and release may be unexpected (11). Inadequate discharge/release planning by prison mental health services has been found to compound the problems associated with transition of care to the community (12, 13). To overcome this challenge The Royal College of Psychiatrist’s Quality Network for Prison Mental Health Services (QNPMHS) has recommended contacting the new care coordinator/service provider within 14 days of release/transfer from prison as a desirable standard of quality of care for Prison Mental Health Services (14, 15). The Cloverhill PICLS service has been a member of the QNPMHS since the inception of the QNPMHS in 2015.

Significant efforts have been made to evaluate the effectiveness and ultimate cost of more assertive efforts at engaging newly released prisoners to identify and reduce potential barriers to community reintegration and improve engagement with necessary health and social services (16, 17). These approaches involve case management in the pre- and post- release periods for varying amounts of time (11). One such model is Assertive Community Treatment (ACT) which has been used to provide intensive case management for up to one year post release (18) and in a more time limited form to improve transfer of care while reducing costs (17). Critical Time Intervention (CTI) also involves the allocation of a case manager in the pre- and post-release period and has been shown to improve engagement with community mental health teams following release from prison (16, 19).

Clinical outcomes for the PICLS Cloverhill multi-year cohorts 2006–2011 (7) and 2012–2014 (8) have been previously been described by the authors. These papers have shown that a relatively small prison mental health team in a busy remand prison can sustainably achieve and measure effective identification of major mental illness and diversion to healthcare, including 26,261 consecutive remands and 4304 patients taken onto PICLS caseloads during the 9 years 2006–2014 (7, 8). This model has enabled mentally ill prisoners to be mapped from the point of identification to that of discharge and referral to receiving services in prison and the community. Referral is not the same as successful transfer of care. Information regarding successful transfer care to non-inpatient community based mental healthcare has not been presented previously in this location.

In the current paper (the third consecutive multi-year cohort in this series of studies) we aimed to address this important issue by determining the proportion of mentally ill people in Ireland’s main remand prison referred to outpatient community mental health and primary care services by PICLS, who successfully achieved transfer of care following their release. For those who did not attend for follow-up, we aimed to identify reasons why this was the case. In addition, we aimed to explore the demographic, clinical, service and offence related factors associated with successful transfer of care. Finally, we aimed to map healthcare outcomes for all other incarcerated individuals discharged by the PICLS team within the study period, namely those who were transferred to inpatient psychiatric settings as well as those discharged to other services within the prison system.

## Methods

This retrospective observational cohort study included all incarcerated persons on remand (pre-trial) seen and discharged by the PICLS team within the three-year study period, 1^st^ January 2015 to 31^st^ December 2017. Upon reception at Cloverhill Prison all newly imprisoned individuals were screened by prison general nursing staff including questions regarding medical and mental health symptoms, previous contact with mental health services and whether or not they had history of self-harm or suicide attempts. All were then assessed by a prison general practitioner within 24 hours of reception. Referrals for mental health assessment by PICLS are made automatically based on this screening and assessment process. Additionally, within one working day of a person’s reception at the prison, PICLS staff reviewed the results of screening and GP assessments, as well as previous electronic clinical case records. If a newly received prisoner was identified as having acute mental needs, they were transferred directly to a landing reserved for vulnerable or mentally ill prisoners, where PICLS aimed to assess them on the following working day. PICLS also accepted referrals from multiple other sources including prison staff, prison psychology, prison addiction counsellors and chaplaincy.

Regarding inclusion criteria, only those remanded at the time of first contact with the PICLS team who were subsequently discharged between 1^st^ January 2015 and 31^st^ December 2017, were included in the current study. Sentenced (non-remand/convicted) individuals were not included.

Decisions regarding clinical urgency and security requirements for proposed hospital admissions were guided during the study period by a validated structured professional judgement instrument, the DUNDRUM Toolkit (9). For those discharged to community settings, detailed referral letters have been prepared for receiving community services since the inception of the service in 2006. PICLS staff also prepare reports for courts and attend court to facilitate diversions to healthcare in the community when required. In the event of transfer to other prison services, the PICLS team similarly provided detailed referral letters to the receiving service. These included to prison general practitioners, prison addiction services and prison inreach mental health teams in sentenced prisons such as that described previously by Smith et al. (20).

### Variables, data sources and measurements

This study was based on routine discharge data collected as part of normal clinical work and service evaluation. This data was finalised at the point of discharge/transfer to enable audit of activity, efficiency and effectiveness of the PICLS service. Where possible, data was recorded in binary format as with previous multi-year descriptions of service activity. No individual patient data is presented. All data was analysed retrospectively and anonymised at the point of completion of the study.

Presence of psychotic or other symptoms was elicited at initial and review clinical interviews by pairs of PICLS team members as part of normal clinical practice for the PICLS team. Active psychotic symptoms were defined as hallucinations, delusions and/or thought disorder. Primary diagnoses were derived by the PICLS team using ICD-10 criteria based on serial clinical interviews and collateral information. Final diagnoses were agreed by the team at the point of discharge or transfer. Homelessness was defined as not having regular accommodation, rough sleeping or residence in homeless shelters at the time of or during committal. Self-harm was defined as a past history of deliberate self-harm, based on patient responses at serial interview and collateral information. Information regarding index offence was gathered at interview and from the Prison Health Information System, as part of usual service practice. Offence type was defined as the most serious offence where there were multiple charges. A violent offence was defined as an act of physical violence on a person and included homicide, assault, robbery, aggravated burglary, contact sexual offences, false imprisonment, driving offences involving injury to others and arson where there was a possibility of injury to others.

Transfer of care was defined as attendance for face-to-face assessment with the receiving mental health service. Information regarding transfer of care was obtained from written correspondence received from the receiving service. Where this was not the case, members of the PICLS team contacted the receiving service by telephone to confirm whether the individual had attended for appointment. If so the date of this first face to face contact was recorded. Successful transfer of care for prison transfers was confirmed using the prison electronic medical records system.

### Ethics

The research protocol for this study was approved by the National Forensic Mental Health Service Research, Audit, Ethics and Effectiveness Committee. Data was routinely collected for service evaluation, completion of annual reports and to demonstrate compliance with standards for the Quality Network for Prison Mental Health Services (Royal College of Psychiatrists) (15). Patients provided written consent to contact their GP and community based mental health services as part of normal clinical activity. Only anonymised data from a large patient sample was analysed and presented.

### Data analysis

Anonymised data was analysed using IBM SPSS (Statistical Software for the Social Sciences) version 29. We used Chi-square tests to determine associations for dichotomous variables and a Fisher Exact test when there was an expected count of less than five in any of the groups. We used *t*-tests to compare continuous variable means between groups. Confidence intervals for proportions were calculated using the Epi-Tools program (21).

## Results

### Mapping

**Figure 1:**
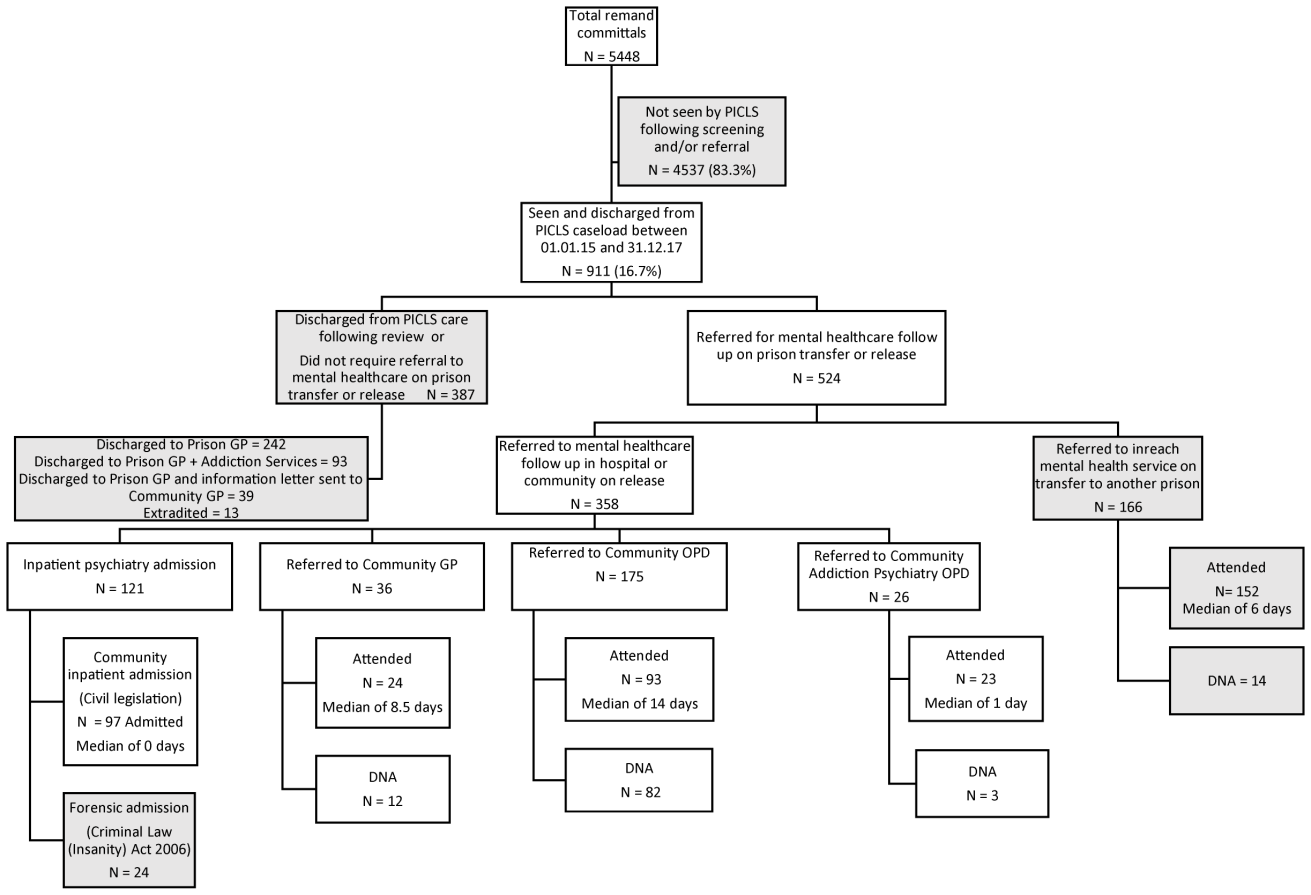
Flow diagram from remand to final treatment arrangements on transfer/release for 911 consecutive male discharges from PICLS Cloverhill, 2015-2017. PICLS, Prison Inreach and Court Liaison Service; GP, General Practitioner, OPD, Outpatient Department; DNA, Did Not Attend.

**Figure 1** shows the path from point of reception to discharge outcome for all 5448 episodes where men remanded to custody in Cloverhill Prison (committal episodes) were discharged from the PICLS caseload from 1^st^ January 2015 to 31^st^ December 2017. Of these, 4537 committal episodes were assessed as not requiring psychiatric assessment following screening and/or referral. The remaining 911 committal episodes were seen, discharged, transferred and diverted by the PICLS service during the three-year study period. These individuals were assessed by the PICLS team a median of three days after the date of their reception at the prison (mean 15.2 days) and each was seen by the PICLS team an average of 4.5 times prior to their discharge from the service.

### Referrals at point of discharge/transfer

**Table 1** shows clinical outcomes and referrals at point of release or transfer for all 911 patients discharged from the PICLS caseload over the three years. Of these, 58% (524/911) were referred for mental health follow up, while the remaining 43% (387/911) were assessed as not requiring mental health care follow up and were discharged to prison general practitioner and/or addiction services. A smaller proportion of these individuals were extradited/deported to another jurisdiction or released directly to the community. For the latter group, the PICLS team sent a letter to their community GP for information purposes only. **Table 1** also shows median and mean times from committal (date of reception at the prison) and from initial mental health assessment to final healthcare outcome at point of discharge or transfer. Median time from committal to discharge was 23 days (mean 52.3 days) and from initial mental health assessment to discharge was 13 days (mean 38.4 days).

**Table 1:** Referral outcomes for all 911 patients seen and discharged by PICLS Cloverhill between 1st January 2014 and 31st December 2017, times from committal to outcome and times from first assessment to outcome.

			Days from committal to outcome				Days from first assessment to outcome			
Outcome	N	%	Median	Range	Mean	SD	Median	Range	Mean	SD
Hospital diversions										
**Forensic hospital admission**	24	2.6	63.5	0-571	107.3	131.5	61.0	0-345	87.4	87.8
**General hospital admission**	97	10.6	25.0	2-139	33.2	29.5	22.0	0-128	29.1	27.7
Prison transfers										
**Prison transfer for MH follow up**	166	18.2	39.5	0-1193	85.8	143.4	28.0	0-1192	75.8	141.8
Community outpatient referrals										
**Community Psych OPD**	175	19.2	23.0	1-395	46.2	61.1	18.0	0-389	38.1	54.8
**Community Addiction Psych OPD**	26	2.8	35.0	1-539	72.2	115.5	26.5	0-535	68.3	115.5
**Community GP**	36	3.9	14.0	2-97	21.7	20.7	10.0	0-97	18.1	21.4
Not referred for mental health follow up										
**Discharged to Prison GP**	242	26.6	18.0	0-726	45.3	84.2	1.0	0-518	20.5	52.8
**Prison GP and addiction**	93	10.2	16.0	1-368	38.3	57.4	0.0	0-366	17.1	45.8
**Community GP (information letter only)**	39	4.2	24.0	3-138	33.9	32.9	14.0	1-138	24.6	29.9
**Extradited/Deported**	13	1.4	36.0	11-205	75.8	72.1	22.0	9-189	66.1	65.6
**Total**	911	100	23.0	0-1193	52.3	90.1	13.0	0-1192	38.4	80.3

GP, General Practitioner; OPD, Outpatient Department.

Primary ICD-10 diagnoses at time of discharge for all 911 patients assessed by PICLS are shown in **Table 2**. Demographic, clinical, service and offence related characteristics for all those seen (N=911) are displayed in **Table 3**. The majority were Irish (86%). The average age at time of first assessment was 32.7 years. Over a third were homeless at the time of reception and 17% received the support of the PICLS housing support service prior to their discharge/release. One third had a diagnosis of bipolar or schizophreniform disorders and 29% presented with active symptoms of psychosis following their committal. Almost 90% had a lifetime history of substance use problems and 59% reported a history of self-harm. Almost one third were charged with a violent offence and the median duration from date of committal to date of disposal was 23 days (mean 52.3 days). **Table 3** also displays a comparison of demographic, clinical, service and offence related characteristics for those referred by PICLS for mental health follow up at the time of discharge (58%, 524/911), with those assessed as not requiring such follow up (43%, 387/911). Those referred for mental health follow up were more likely to be older at the time of initial assessment, to have been homeless at the time of reception, and to have received support from the PICLS Housing Support Service. They were also more likely to have been charged with a non-violent offence.

**Table 2:** Primary ICD-10 diagnosis at point of discharge for 911 incarcerated individuals seen and discharged from PICLS Cloverhill from 2015-2017, with diagnoses for those referred to mental health follow up in hospital, prison and community settings, and for those not referred for mental health follow-up.

		All those seen and discharged by PICLS 4 N=911 (100%)		Not referred for Mental Health follow-up N=387 (42.5%)		Referred for Mental Health follow-up N=524 (57.2%)					
						Any Hospital Admission (N=121)		Other Prison MHS (N=166)		Community OPD and Primary Care (N=237)	
ICD-10 Code	Primary ICD-Diagnosis	N	%	N	%	N	%	N	%	N	%
**F00-09**	Organic disorders	15	1.6	4	1.0	1	0.8	5	3.0	5	2.1
**F10-19**	Substance Use Disorders	302	33.2	216	55.8	2	1.7	37	22.3	47	19.8
**F20-29**	Schizophreniform Disorders	261	28.6	9	2.3	95	78.5	61	36.7	96	40.5
**F30-39**	Mood Disorders	98	10.8	31	8.0	20	16.5	13	7.8	34	14.3
**F40-59**	Neurotic Disorders	17	1.9	14	3.6	0	0	1	0.6	2	0.8
**F60-69**	Personality disorders	148	16.2	78	20.2	0	0	34	20.5	36	15.2
**F70-79**	Mental Retardation	14	1.5	3	0.8	0	0	4	2.4	7	3.0
**F80-98**	Developmental/Childhood Disorders	27	3.0	7	1.8	3	2.5	8	4.8	9	3.8
	No Mental illness diagnosed	29	3.2	25	6.5	0	0	3	1.8	1	0.1
**Total**		911	100	387	100	121	100	166	100	237	100

PICLS, Cloverhill Prison Inreach and Court Liaison Service; MHS, Mental Health Service; OPD, Outpatient Department.

**Table 3:** Clinical and demographic descriptors for all those seen and discharged (N = 911) from PICLS Cloverhill during the period 2015-2017, and comparison of those referred for mental health follow up, with those who were not.

	Total Discharges N = 911 (100%)			Referred for mental health follow up N=524 (57.5%)			Not Referred for mental health follow up N=387 (42.5%)			Statistical test of difference	*P* -value
	N (%)	Mean (SD)	Median	N (%)	Mean (SD)	Median	N (%)	Mean (SD)	Median		
**Age**		32.7 (10.9)	31.0		33.5 (11.2)	32.0		31.5 (10.5)	29.0	t *=* 2.89	0.04
**Nationality - Irish**	786 (86.3)			452 (86.3)			334 (86.3)			*X^2^* ***=*** 0.00	0.98
**Active Psychotic Symptoms following committal**	267 (29.3)			250 (47.7)			17 (4.4)			*X^2^* ***=*** 201.6	<0.001
**ICD10 F20-F31 Diagnosis**	304 (33.4)			290 (55.3)			14 (3.6)			*X^2^* ***=*** 267.9	<0.001
**Homeless**	342 (37.5)			231 (44.1)			111 (28.7)			*X^2^* ***=*** 22.5	<0.001
**Seen by Housing Support Service during committal**	161 (17.7)			134 (25.6)			27 (7.0)			*X^2^* ***=*** 53.1	<0.001
**Lifetime history of Substance Use Problems**	818 (89.8)			475 (90.6)			344 (88.9)			*X^2^* ***=*** 0.8	0.4
**Lifetime History of Self-Harm**	538 (59.1)			290 (55.3)			248 (64.2)			*X^2^* ***=*** 7.3	0.01
**Violent Index Offence (current)**	280 (32.7)			136 (26.0)			144 (37.2)			*X^2^* ***=*** 13.3	<0.001
**Duration from date of committal to date of disposal (days)**		52.5 (90.1)	23.0		58.7 (99.7)	27.5		43.5 (74.4)	18.0	t *=* 2.64	0.08
**Times seen by the PICLS team**		4.5 (5.9)	2.0		6.0 (6.9)	4.0		2.4 (2.8)	1.0	t *=* 10.7	<0.001

PICLS, Cloverhill Prison Inreach and Court Liaison Service; SD, Standard Deviation.

From a clinical perspective those referred for mental health follow up were more likely to present with active psychotic symptoms following committal and to be diagnosed with ICD-10 diagnosis of a schizophreniform or bipolar affective disorder (ICD-10 F20-F31). Of note 14 patients, representing 4.6% of those with a primary ICD-10 diagnosis of F20-F31 were not referred for mental health follow up at the time of discharge by PICLS. These included patients who were released and a letter forwarded to their GP for information purposes, patients discharged to the prison GP and/or addiction services and seven who were extradited/deported. There was no significant difference in the rates of substance use problems or duration from committal to discharge between the two groups. Patients referred for mental health follow-up had been seen an average of 4.5 occasions (SD 5.9, range 1–50) by pairs of PICLS team members. Those referred for mental health follow up on discharge/transfer had been seen an average of six occasions, compared with an average of 2.4 times for those not so referred.

### Referral outcomes and transfer of care - hospital admissions, prison transfers and referrals to community outpatient services

For all those referred for mental health follow up, 79% (414/524) achieved transfer of care at the receiving hospital, prison or community outpatient or primary care setting.

### Hospital admissions - Diversions to forensic and community inpatient settings

**Table 1** shows that 13% of patients (121/911) were admitted to psychiatric hospitals from remand. Overall, under 3% (24/911) were admitted to the Central Mental Hospital, Ireland’s only forensic hospital at the time, under the Criminal Law (Insanity) Act 2006, while over 10% (97/911) were diverted to community inpatient psychiatric hospital settings throughout Ireland under civil legislation, the Mental Health Act 2001. Of those referred for mental health follow up, 23% (121/524) were admitted to psychiatric hospitals, with 5% admitted to the forensic hospital and 19% admitted to local general psychiatric hospitals. **Table 2** shows that almost 80% of hospital admissions (95/121) were of patients with a primary diagnosis of schizophreniform disorders (ICD-10 F20-F29).

### Prison transfers - referrals to other prison inreach mental health services

The PICLS team referred 166 patients to mental health colleagues in other prisons on sentencing or other prison transfer, representing almost one third (32%, 166/524) of all those referred for mental health follow-up. The primary ICD-10 diagnoses at the time of discharge are shown in **Table 2**, including over one third with a primary ICD-10 diagnosis of a schizophreniform disorder (61/166, 37%).

**Table 4** displays the outcomes for these referrals along with timeframes to first face-to-face assessment in the receiving prison or failure to achieve successful transfer of care. Over 90% of prison transfers (92%, 152/166) were seen by the receiving prison inreach mental health service following their discharge from PICLS. The median duration to follow-up was six days with a range of 0–366 days. The remaining 14 prison transfers who were not seen by the receiving inreach mental health service either did not attend their scheduled appointment or were not accepted by the receiving service.

**Table 4:** Transfer of care (TOC) outcomes and timeframes for 237 patients discharged from PICLS Cloverhill to community outpatient and primary care settings (N=237) and other Prison Mental Health Services (N=166), 2015-2017.

Outcome	TOC achieved					TOC not achieved		
	N	%	95%CI	Median (days)	Range (days)	N	%	95%CI
**All Community outpatient referrals (N = 237)**	140	59.1	52.2-65.4%	9 (7 omitted as no date available)	0-459	97	40.9	34.6-47.5%
**Referrals to Community General Psychiatry OPD (N = 175)**	93	53.1	45.5-60.7%	14 (3 omitted as no date available)	0-378	82	46.9	39.3-54.5%
**Referrals to Community Addiction Psychiatry OPD (N = 26)**	23	88.5	69.9-97.6%	1	0-365	3	11.5	2.5-30.2%
**Referrals to Community Primary Care for mental health follow up (N = 36)**	24	66.7	49.0-81.4%	8.5 (4 omitted as no date available)	0-459	12	33.33	18.6-50.9%
**Referrals to other Prison MHS (N=166)**	152	91.6	86.3-95.3%	6	0-366	14	8.4	4.7-13.8%

PICLS, Cloverhill Prison Inreach and Court Liaison Service; MHS, Mental Health Service; OPD, Outpatient Department; TOC Transfer of Care defined as face-to face meeting with community primary care or psychiatry outpatient service following release from prison.

Demographic, clinical, service and offence related characteristics for this group are displayed in **Table 5**. Prison transfers for whom successful transfer of care was achieved were younger, had spent longer periods on remand and had more documented assessments by the PICLS team prior to transfer than those for whom successful transfer of care was not achieved. Those who achieved transfer of care did not differ significantly in terms of nationality, presence of active psychotic symptoms, diagnosis, housing status, self-harm history or offence type (violent/non-violent), from those for whom successful transfer of care was not achieved.

**Table 5:** Prison Transfers: Comparison between clinical and demographic variables of all 166 male remand patients referred on transfer to other prisons from PICLS Cloverhill from 2015 – 2017 for whom successful transfer of care was and was not achieved.

	All committals referred to another prison MHS on transfer from Cloverhill Prison N=166 (100%)			Transfer of Care achieved N= 152 (91.6%)			Transfer of Care NOT achieved N=14 (8.4%)			Statistical test of difference	p - value
	N (%)	Mean (SD)	Median	N (%)	Mean (SD)	Median	N (%)	Mean (SD)	Median		
**Age**		31.5 (9.9)	30.0		30.8 (9.3)	30.0		38.9 (13.2)	36.0	t *=* -2.2	0.04
**Nationality - Irish**	147 (88.6)			135 (88.8)			12 (85.7)			FET	0.7
**Active Psychotic Symptoms following committal**	55 (33.1)			53 (34.9)			2 (14.3)			FET	0.1
**ICD10 F20-F31 Diagnosis**	70 (42.2)			66 (43.4)			4 (28.6)			*X^2^* ***=*** 1.2	0.3
**Homeless**	53 (31.9)			50 (32.9)			3 (21.4)			FET	0.6
**Seen by Housing Support Service during committal**	26 (15.7)			25 (16.4)			1 (7.1)			FET	0.7
**Lifetime history of Substance Use Problems**	154 (92.8)			142 (93.4)			12 (89.7)			FET	0.3
**Lifetime History of Self-Harm**	104 (62.7)			98 (64.5)			6 (42.9)			*X^2^* ***=*** 2.56	0.1
**Violent Index Offence (current)**	69 (41.6)			65 (42.8)			4 (28.6)			*X^2^* ***=*** 1.1	0.3
**Duration from date of committal to date of prison transfer (days)**		85.8 (143.4)	39.5		90.9 (148.5)	48.5		30.0 (32.3)	15.0	t *=* 4.1	<0.001
**Times seen by the PICLS team**		6.0 (8.4)	3.0		6.4 (8.7)	3.0		2.4 (2.2)	1.5	t *=* 4.4	<0.001

PICLS, Cloverhill Prison Inreach and Court Liaison Service; FET, Fisher’s Exact Test; SD, Standard Deviation.

### Community mental health outpatient referrals - referrals to community outpatient psychiatric clinics or primary care services

The PICLS team referred 237 patients to community outpatient settings on release from custody over the three years. This represented 26% of all 911 incarcerated individuals discharged from the PICLS caseload during this period and 45% of the 524 referred for mental health follow up by the PICLS team.

**Table 4** shows that 85% (201/237) of referrals for community follow up were made to community mental health outpatient clinics (N=175) or addiction psychiatry (service for patients with a major mental illness on methadone maintenance therapy) outpatient clinics (N=26). The remaining 15% were referred for mental health follow up with community primary care services. **Table 6** shows demographic, clinical, service and offence related characteristics for all 237 patients referred for community based mental health care follow-up on release. Of these, 91% were of Irish nationality. These included 6% (14/237) who identified as being from an Irish Traveler background. For those remaining, 5% (11/237) were EU non-Irish, and 5% (11/237) were from a non-EU background. The mean age at time of first assessment was 33.4 years. Almost half of all those seen were homeless at the time of initial assessment by the PICLS team.

**Table 6:** Community OPD and Primary Care Referrals: Association between clinical and demographic variables of those discharged from PICLS Cloverhill from 2015 – 2017 and successful transfer of care (N = 237).

	All committals referred to community OPD or primary care on release N = 237 (100%)			Transfer of Care achieved N=140 (59.1%)			Transfer of Care NOT achieved N=97 (40.9%)			Statistical test of difference	p - value
	N (%)	Mean (SD)	Median	N (%)	Mean (SD)	Median	N (%)	Mean (SD)	Median		
**Age**		33.4 (11.1)	32.0		33.4 (10.1)	33.0		33.5 (12.5)	31.0	t *=* -0.1	0.9
**Nationality - Irish**	215 (90.7)			126 (90.0)			89 (91.8)			*X^2^* ***=*** 0.21	0.6
**Active Psychotic Symptoms following committal**	83 (35.0)			46 (32.9)			37 (38.1)			*X^2^* ***=*** 0.7	0.4
**ICD10 F20-F31 Diagnosis**	107 (45.1)			66 (47.1)			41 (42.3)			*X^2^* ***=*** 0.55	0.5
**Homeless**	117 (49.4)			73 (52.1)			44 (45.4)			*X^2^* ***=*** 1.05	0.3
**Seen by Housing Support Service during committal**	79 (33.3)			54 (38.6)			25 (25.8)			*X^2^* ***=*** 4.22	0.04
**Lifetime history of Substance Use Problems**	219 (92.4)			129 (92.1)			90 (92.8)			*X^2^* ***=*** 0.03	0.9
**Lifetime History of Self-Harm**	140 (59.1)			80 (57.1)			60 (61.9)			*X^2^* ***=*** 0.53	0.5
**Violent Index Offence (current)**	34 (14.3)			20 (14.3)			14 (14.4)			*X^2^* ***=*** 0.001	1.0
**Duration from date of committal to date of release (days)**		45.3 (66.3)	21.0		43.3 (54.5)	23.5		48.2 (80.5)	21.0	t *= -*0.5	0.6
**Times seen by the PICLS team**		4.9 (5.4)	2.0		4.9 (5.4)	2.0		5.0 (5.5)	2.0	t *=* -0.1	0.9

PICLS, Prison Inreach and Court Liaison Service; OPD, Outpatient Department; SD, Standard Deviation.

**Table 2** shows primary ICD-10 diagnoses at date of discharge for all 237 patients referred to outpatient community mental health and primary care services during the study period. **Table 6** shows that 45% (107/237) were diagnosed with bipolar or schizophreniform disorder. Over 90% had a lifetime history of polysubstance abuse and 59% a lifetime history of self-harm. Over one third of those discharged to community mental health and primary care services presented with evidence of active psychotic symptoms while in custody. Of these, 72% received a primary diagnosis of a schizophreniform disorder, 8% a mood disorder and 19% a substance induced psychotic episode. Over 85% (203/237) of those referred to outpatient community mental health and general practitioner services had been charged with a non-violent offence.

**Table 4** shows the outcomes for these referrals along with timeframes to first face-to-face assessment by community mental health or primary care services, or failure to achieve successful transfer of care. Of the 237 patients referred, 59% were confirmed as having attended the receiving service at any time following their release. The median duration to follow up was 9 days, and 7 cases were omitted from the analysis of timeframes as a specific date was unavailable. Over half of those referred to community mental health outpatient clinics (53%, 93/175) attended following release, with a median of 14 days and a range of 0–378 days. Three cases were omitted from this analysis because the receiving service confirmed attendance, but did not provide a specific date. Forty one percent (97/237) of those referred to community mental health and primary care services did not attend following release, 85% (82/97) of who were referred to general adult community mental health outpatient clinics. Of these: 53 (64%) did not attend their scheduled outpatient appointment; in 12 cases the community mental health team declined the referral and for 17 cases the outcome was unknown as the receiving service did not provide any information. Of those referred to community addiction psychiatry clinics, 89% (23/26) attended in a median of one day (one case omitted as no information was provided by the receiving service). Two thirds (24/36) of those referred for primary care follow up were confirmed as having attended in a median of 8.5 days, with nine either not attending or not being offered an appointment, and three cases were omitted as no specific date of attendance was provided by the receiving GP practice.

Demographic, clinical, service and offence related characteristics for referrals to community primary care and community mental health settings are displayed in **Table 6**. Referrals to community care settings for whom successful transfer of care was achieved did not differ significantly from those for who did not attend, in terms of mean age, presence of psychotic symptoms, ICD-10 diagnosis F20–31 while on remand, nationality, housing status, self-harm history, offence type (violent/non-violent), time on remand or number of reviews by the PICLS team. A significantly greater proportion of patients who achieved successful transfer of care had received the assistance of the PICLS Housing Support Worker during the remand period compared with those who did not achieve successful transfer of care.

## Discussion

### Summary of findings

This is one of a series of papers “counting in and counting out” all persons remanded to Cloverhill Prison since the inception of the PICLS service in 2006, involving 31,709 remand committal episodes as at 31^st^ December 2017. We have previously described a “STRESS-Testing” model to assess the effectiveness and efficiency of our service over time, and to enable international benchmarking (8). That paper summarised caseloads and clinical outcomes, but noted as a limitation that arrangements for transfer of care are not the same as achieving health gains. Given that previous research had described poor rates of engagement with general psychiatry services in the community following release from prison (12), it was noted that the next paper in this series should present outcome data for community outpatient diversions.

In this retrospective observational study, we have shown that the majority of incarcerated individuals (79%, 414/524) assessed by PICLS as requiring mental health follow up upon discharge or transfer achieved transfer of care at the receiving hospital, prison, community outpatient or primary care setting. Receiving support from the team’s housing support service was associated with successful transfer of care to community based mental health and primary care services.

For those patients referred to other prison inreach teams in Ireland on sentencing or other prison transfer during the three-year study period, 91.6% achieved successful transfer of care, with a median time to first face-to-face contact of nine days.

### Strengths and limitations

Previous studies have identified difficulties in successfully transferring the care of patients with mental illness from prison settings, particularly remand prisons, to mental health services in the community (12, 13). In the current study, despite all participants being on remand and spending a median of just 21 days in custody (mean 45.3 days SD 66.3), 59.1% of those patients released directly to the community achieved transfer of care, with 41.8% doing so within six weeks of release and referral. The systematic approach taken by the PICLS service to identify mentally ill men as soon as possible after reception at the prison, and to arrange for health care follow up through early liaison with the receiving mental health service (8) may have contributed to its relative success in achieving transfer of care. The PICLS model embodies some of the key practices identified as improving outcomes for those transitioning from prison to the community (17, 22). Similar to the practices outlined by prison staff in a study by Hancock et. al., the PICLS model included clearly defined and effective communication pathways and provided support in obtaining accommodation prior to the person’s release (22). The systematic approach taken by PICLS in identifying incarcerated individuals with mental disorders and arranging for appropriate health care follow up with the provision of time limited post-release support, reflects the positive impact described by McKenna et al. (17) following the implementation of an assertive community treatment informed prison in-reach model of care (PMOC). This prison in-reach model of care resulted in a significant improvement in the rate of released prisoners taking up at least one face-to-face contact with community mental health services.

The addition of a housing support service to the PICLS model, has enhanced the service’s interagency approach to release planning. This was confirmed in the current study which determined that those transitioning to community based mental health supports, were more likely to achieve successful transfer of care if supported by the PICLS housing support service. This pragmatic support in the pre- and post-release period may act as an incentive for remand prisoners to engage with mental health supports upon release. This finding is consistent with the findings of the RESET study (23), which identified similar positive links between support with accommodation on release from custody and subsequent engagement with mental health follow up.

The PICLS Housing support service did not offer intensive case management post-release, equivalent to Assertive Community Treatment (ACT) or Critical Time Intervention (CTI) however it did provide practical supports in the pre- and post-release period, by contributing to the development of holisitic pre-release plans and providing time-limited support post-release for some patients, including accompanying released prisoners to their housing locations and first mental health appointment. Previous studies (19, 22) and a systematic review (11) have highlighted the importance of pre-release planning, which was one of the key roles of this service. There is a significant drive in NHS England and NHS Improvement (UK) to support prison leavers to engage with services. RECONNECT is a care after custody service, which aims to support prison leavers to remain engaged with appropriate treatment upon resettlement, through referrals to community-based health services (24). Hunter et al. conducted an economic evaluation of a complex intervention (Engager) for prisoners with common mental health problems, near to and after release (25). The consequences component of the cost-consequences analysis provided some evidence for increased access to services such as substance misuse services, but it also showed an increase in unplanned service use, particularly for specialist mental health services (noting that only one mental health service was identified as planned). There was no evidence that the Engager intervention was cost-effective compared to usual care.

### Limitations

The sample consisted of male remands only. The study was not a full national sample, but did describe a full mental health caseload sample over an extended period for the country’s main remand prison, receiving a majority of remands nationally. Countries with full national databases may be able to provide more comprehensive data. The study focussed on patient-related factors and factors related to our service. It did not take account of community service-related factors which might influence attendance outcomes.

As the study was naturalistic, describing actual practice for all patients on the PICLS caseload, there was no control group and the results may not be generalisable to other services or jurisdictions. Nonetheless as we have previously described outcome data over time in the same location, outcomes other than the successful transfer of care for referrals to prison and community outpatient settings can be compared with the previous extended timeframes since 2006. The 24 admissions to a forensic hospital under the Criminal Law (Insanity) act 2006 over three years represents a sharp reduction in numbers and as a proportion of the caseload compared with the previous three-year sample in this series, while admissions achieved to general psychiatric hospitals under civil legislation increased. We have not described what became of people placed on waiting lists for hospital admission but not achieving such admission in this paper, but have done so in a separate paper describing a full national sample over the five-year period 2015–2019 (10).

This higher rate of successful transfer of care to prison mental health services in comparison to community settings may be related to service and jurisdiction specific factors which may not be generalisable elsewhere. During the timeframe studied, prison in-reach mental health teams were provided by the National Forensic Mental Health Service to all but two of Ireland’s prisons in the south-west of the country (Cork and Limerick), where prison mental health services were provided by local mental health services. This centralised approach may have contributed to closer working relationships between mental health teams throughout the prison network. Team members attended regular governance meetings convened by the National Forensic Mental Health Service to discuss caseloads and referrals. All transfers between prisons for people on mental health caseloads were followed by an email to the receiving team sent via team administrators at the National Forensic Mental Health Service. In addition, all Irish prisons shared a single electronic medical records system, accessible from all prison locations further enhancing communication between prison in-reach mental health teams.

We do not have data in this paper on the proportion of people who were supported by Probation services on release to the community. The majority of community diversions involved people released on bail rather than use of the Probation Acts. Finally, the current study did not include any information on rates of reimprisonment or reoffending. This may be important because a previous systematic review exploring the impact of interventions aimed at improving continuity of care for mentally ill prisoners as they return to the community unexpectedly found increased rates of reimprisonment among those receiving such interventions. Future studies will aim to address whether or not this was the case for our service.

### Service implications and future directions

The positive association between contact with our housing support service and engagement with mental health services following release from custody is an important finding and may have implications for service planning. During the study period there was a single housing support worker assigned to the PICLS team, showing that a relatively low-cost intervention can have far-reaching positive outcomes beyond the relatively narrow remit of their role. This strengthens the recommendation made by a recent Irish governmental High Level Task Force to expand the PICLS services including its housing support service to other committal prisons in Ireland (26).

In conclusion, this study shows that the PICLS model for identifying mental illness in a remand prison setting and arranging appropriate mental health care follow upon release from custody was sustainable over time and did not involve major resource implications. It shows that the majority of individuals attended their scheduled mental healthcare appointments on release. However, the sustainable, efficient and safe diversion of patients with severe mental illness from a prison setting by a team such as PICLS requires access to appropriate levels of psychiatric inpatient beds within both general adult psychiatry and forensic psychiatry settings in order to succeed. There is scope for expanding the PICLS service nationally and in particular for increasing the provision of Housing Support Workers, particularly for remand settings. While the proportion achieving successful transfer of care to community outpatient settings on release was less than for prison transfers, this compared well with previous studies in other jurisdictions. Systematic communication and the assistance of a Housing Support Worker were particularly beneficial. Enhanced development of, and integration with community assertive outreach services in community settings would be expected to further improve the proportion of patients with mental illness achieving successful transfer of care on release from custodial settings. Enhanced co-working with Probation Service would also be of benefit as would more specific legislation to enable community treatment orders with supervision provisions.

## Data availability statement

The raw data supporting the conclusions of this article will be made available by the authors, without undue reservation. Requests to access these datasets should be directed to cjoneill@irishprisons.ie.

## Ethics statement

The studies involving humans were approved by National Forensic Mental Health Service, Dundrum, Dublin, Ireland. The studies were conducted in accordance with the local legislation and institutional requirements. Written informed consent for participation was not required from the participants or the participants’ legal guardians/next of kin in accordance with the national legislation and institutional requirements.

## Author contributions

JW: Formal analysis, Investigation, Writing – original draft, Writing – review & editing, Data curation. DS: Data curation, Formal analysis, Investigation, Methodology, Validation, Writing – original draft, Writing – review & editing. FB: Investigation, Writing – review & editing. PH: Investigation, Writing – review & editing. ET: Investigation, Writing – review & editing. MC: Investigation, Writing – review & editing. OR: Investigation, Writing – review & editing. CO: Conceptualization, Data curation, Formal analysis, Investigation, Methodology, Supervision, Validation, Writing – original draft, Writing – review & editing.
